# Expression of unfolded protein response markers in the pheochromocytoma with Waardenburg syndrome: a case report

**DOI:** 10.1186/s12902-020-00574-9

**Published:** 2020-06-22

**Authors:** Shuhei Morita, Ken Takeshima, Hiroyuki Ariyasu, Yasushi Furukawa, Shohei Kishimoto, Tomoya Tsuji, Shinsuke Uraki, Hiroyuki Mishima, Akira Kinoshita, Yuichi Takahashi, Hidefumi Inaba, Hiroshi Iwakura, Hiroto Furuta, Masahiro Nishi, Asako Doi, Shin-ichi Murata, Koh-ichiro Yoshiura, Takashi Akamizu

**Affiliations:** 1 The First Department of Medicine, Wakayama Medical University, 811-1 Kimi-idera, Wakayama, Wakayama 641-8509 Japan; 2 Department of Human Genetics, Atomic Bomb Disease Institute, Nagasaki University, Nagasaki, Japan; 3 Department of Diagnostic Pathology, Wakayama Medical University, Wakayama, Japan

**Keywords:** Pheochromocytoma, Unfolded protein response, Endoplasmic reticulum, Waardenburg syndrome, RET, Case report

## Abstract

**Background:**

It is clinically emergent to further understand the pathological mechanism to advance therapeutic strategy for endocrine tumors. A high amount of secretory protein with tumorigenic triggers are thought to induce unfolded protein response in endoplasmic reticulum in endocrine tumors, but its evidence is limited.

**Case presentation:**

A 40-year-old woman had an approximately 10-year history of intermittent headaches. After the incidental detection of a mass in her right adrenal gland by CT scan, she was admitted to our hospital. She had been diagnosed as type 1 Waardenburg syndrome with the symptoms of dystopia canthorum, blue iris, and left sensorineural hearing loss. Urinary catecholamine levels were markedly elevated. ^123^I-MIBG scintigraphy showed uptake in the mass in her adrenal gland. After the adrenalectomy, her headaches disappeared and urinary catecholamine levels decreased to normal range within 2 weeks. Genome sequencing revealed germline mutation of c.A175T (p.Ile59Phe) in transcription factor PAX3 gene and somatic novel mutation of c.1893_1898del (p. Asp631_Leu633delinsGlu) in proto-oncogene RET in her pheochromocytoma. RNA expression levels of RET were increased 139 times in her pheochromocytoma compared with her normal adrenal gland. Those of unfolded protein response markers, Bip/GRP78, CHOP, ATF4, and ATF6, were also increased in the pheochromocytoma.

**Conclusion:**

We report a rare case of pheochromocytoma with type 1 Waardenburg syndrome. This is the first case to show the activation of unfolded protein response in the pheochromocytoma with the novel somatic mutation in RET gene. Our findings may support that unfolded protein response is activated in endocrine tumors, which potentially could be a candidate of therapeutic target.

## Background

Although surgical removal is the first line treatment in most of the endocrine tumors including pheochromocytoma, therapeutic alternatives are limited. Further understanding of pathological background in order to advance therapeutic strategy is critical.

Waardenburg syndrome (WS) is an autosomal dominant condition associated with the abnormalities of neural crest cells. It is clinically characterized by sensorineural deafness and by pigment abnormalities [1, 2]. The endocrinological diseases occurring with WS is rare. Kallmann syndrome and hypogonadotropic hypogonadism have been reported as the endocrinological diseases concomitant with WS [3, 4].

Genetic mutation is one of the causes of pheochromocytoma. Mutation of proto-oncogene RET is known to have tumorigenic effect by enhancing kinase receptor signaling [5]. The mutations of the cysteine-rich domain in RET is frequently shown in multiple endocrine neoplasia type 2A (MEN2A) or familial medullary thyroid carcinoma (FMTC) [5]. In most cases with MEN2 or FMTC, the type of mutation is gain of function.

On demand, cells can regulate the protein-folding capacity in the endoplasmic reticulum (ER) [6]. The response to the burden of unfolded proteins in ER lumen is termed the unfolded protein response (UPR). In endocrine cells, a high amount of secretory proteins induces the load on ER as proinsulin does in β-cells in diabetes [7]. Besides there being excessive secretory proteins, tumorigenic factors, such as (proto-)oncogene expression or loss of the tumor suppressors, are thought to be further triggers to overload the ER in endocrine tumors.

Here, we present a patient with pheochromocytoma with WS. Genetic analysis revealed novel somatic mutation of proto-oncogene RET under the germline mutation of paired box gene 3 (PAX3) gene, the encoding protein of which positively regulates RET mRNA expression as a transcription factor [1, 8]. We demonstrate extreme increases in RET mRNA expression and UPR markers in her pheochromocytoma compared to those in her normal adrenal gland. Endogenous overload of secretory proteins combined with oncogenic mutation might be related to activation of UPR in pheochromocytoma with WS.

## Case presentation

**Fig. 1:**
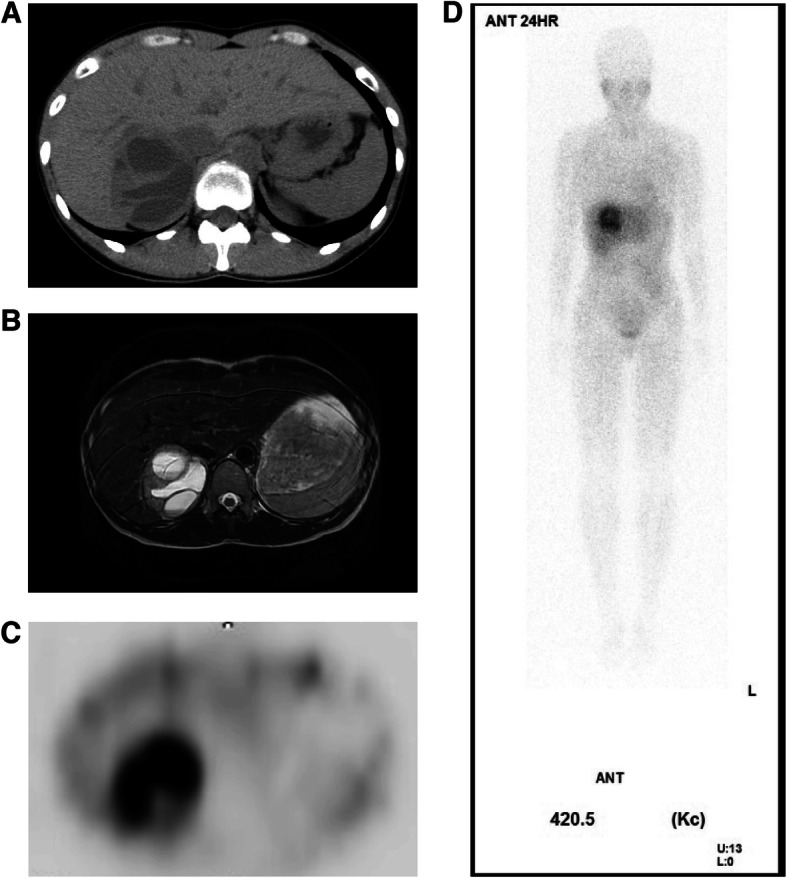
**a** Abdominal computed tomography (CT) shows a mass with cystic components in the right adrenal gland. **b** MRI shows modestly high intensity with cystic components in T2-weighted image. **c** and **d** In ^123^I-MIBG scintigram, increased trace uptake was observed in the right adrenal gland mass

A 40-year-old woman had around 10-year history of intermittent headaches, the frequency of which had been especially increased in recent months. After incidental detection of a mass in her right adrenal gland by abdominal computed tomography (CT) scan, she was admitted to our hospital. She had dystopia canthorum, blue iris, and left sensorineural hearing loss and WS was diagnosed when she was around 5 years old. She had no muscle weakness nor anomaly of her limbs. There was no relevant family history. Laboratory findings including hormonal data are shown in Table 1. Urinary catecholamine and metanephrine levels were markedly elevated, as shown in Table 2. Twenty-four-hour blood pressure profile revealed a hypertensive spike associated with the headaches. Abdominal CT showed a heterogeneous mass enlarged to 62 × 35 mm in size with a cystic component in her right adrenal gland (Fig. 1a). Magnetic resonance imaging (MRI) revealed a heterogeneous mass with cystic component with moderately intensity in T2-weighted image the same size as in the CT image (Fig. 1b). ^123^I-MIBG showed increased uptake in approximately the same area as the right adrenal gland mass (Fig. 1c and d). After blood pressure was controlled with Doxazosin, right adrenalectomy was performed. Two weeks after the operation, her symptoms had disappeared and urinary catecholamine and metanephrine levels were normalized.

**Table 1:** Laboratory Examination

Blood Cell/Biochemistry		Endocrinology	
WBC	4870 /μL	GH	0.2 ng/mL
Hb	12 g/dL	IGF-I	137 ng/mL
PLT	38.7 ×104/μL	PRL	15.5 ng/mL
Alb	4.3 g/dL	LH	1.3 mIU/mL
CK	52 IU/L	FSH	1.5 mIU/mL
AST	21 IU/L	E2	213.4 pg/mL
ALT	26 IU/L	TSH	1.63 μIU/mL
γ-GTP	77 IU/L	FT3	2.71 pg/mL
Cr	0.75 mg/dL	FT4	1.04 ng/dL
BUN	9 mg/dL	ACTH	43.6 pg/mL
UA	3.1 mg/dL	F	12.9 μg/mL
FPG	107 mg/dL	NA	0.39 ng/mL
HbA1c	6.3 %	A	0.22 ng/mL
T-Cho	205 mg/dL	DA	<0.01 ng/mL
TG	49 mg/dL	PAC	7.3 ng/dL
HDL-C	89 mg/dL	PRA	1.7 ng/mL/hr
LDL-C	100 mg/dL	Calcitonin	1.84 pg/mL
Na	137 mEq/L	CEA	2.5 ng/mL
K	3.9 mEq/L	Tg	9.1 ng/mL
		TPO	Ab 9.6 IU/mL
		Tg Ab	<10.0 IU/mL

**Table 2:** 24 h Urinary Examination

	1st	2nd	Reference Range
CA 3F			
NA (μg/day)	259.6	229.6	31.0–160.0
A (μg/day)	309.1	299.4	3.0–41.0
D (μg/day)	910.5	917.2	280.0–1100.0
NM/M 2F			
NM (mg/day)	4.37	4.46	0.10–0.28
M (mg/day)	6.05	6.33	0.04–0.18

Whole exon genome sequence revealed germline heterozygous mutation of c.A175T (p.Ile59Phe) in PAX3 gene and somatic heterozygous mutation of c.1893_1898del (p. Asp631_Leu633delinsGlu) in RET gene in her pheochromocytoma. These mutations were confirmed by Sanger sequencing. The mutation in RET gene detected in the pheochromocytoma was not detected in her normal adrenal gland, which we used as a negative control in analysis of RNA expression. The germline mutation in PAX3 gene was not detected in her mother nor younger brother.

A histological examination of the resected tumor confirmed the diagnosis of pheochromocytoma. The tumor showed characteristic Zellballen architecture. The tumor cells have larger nucleus and more chromaffin granules than normal chromaffin cells. Immunostaining for S-100 protein demonstrates the sustentacular framework surrounding the tumor cells. Immunostaining for synaptophysin, chromogranin A, and CD56 proteins were positive in tumor cells. The MIB-1 index was 1%.

**Fig. 2:**
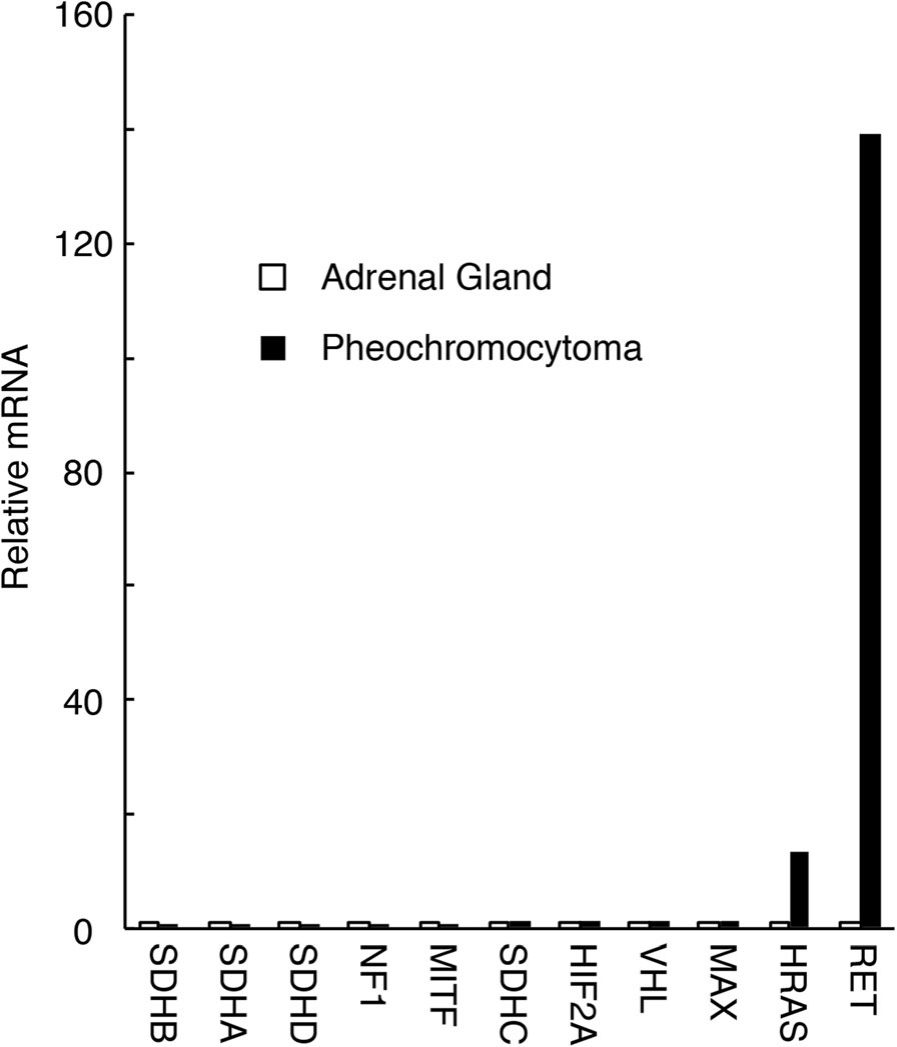
Relative mRNA levels of the indicated mRNAs in the pheochromocytoma with Asp631_Leu633delinsGlu mutations in RET gene to the right normal adrenal gland without those mutations as a control

**Fig. 3:**
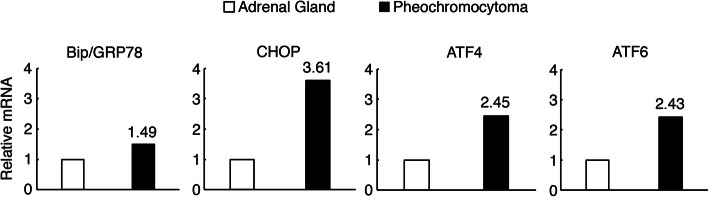
Relative mRNA levels of the indicated mRNAs in the pheochromocytoma with Asp631_Leu633delinsGlu mutations in RET gene to the right normal adrenal gland without those mutations as a control. The numbers indicate the relative mRNA expression levels in the pheochromocytoma to the right normal adrenal gland

RNA expression levels of RET gene with other pheochromocytoma-related genes and UPR markers, Bip/GRP78, CHOP, ATF4, and ATF6, in her pheochromocytoma relative to her normal adrenal gland were analyzed by real time PCR (RT-PCR). RNA was extracted, reverse transcribed, and quantified as we previously reported [9]. Gene expression levels were normalized to β-Actin [9]. Primers used for RT-PCR are shown in Table 3. RET mRNA expressions in the patient’s pheochromocytoma were increased 139 times more than in her normal adrenal gland (Fig. 2). Furthermore, its UPR markers were unexpectedly increased around 1.49–3.61 times more in her pheochromocytoma than her normal adrenal gland (Fig. 3).

**Table 3:** Forward and Reverse Sequences for the Primers Used for RT-PCR Gene

Gene	Primer	
SDHB:	F:	5′-GGAAGGCAAGCAGCAGTATC-3′
	R:	5′-AGCGATAGGCCTGCATAAGA-3′
SDHA:	F:	5′-ACACAGACCTGGTGGAGACC-3′
	R:	5′-CAAAGGGCTTCTTCTGTTGC-3′
SDHD:	F:	5′-CTGGACTAGCGAGAGGGTTG-3′
	R:	5′-CCCAGCAAAGGTTAAAGCTG-3′
NF1:	F:	5′-CACGCAGGTTTTTCCTTGAT-3′
	R:	5′-GAGGACCCAGGTATGCAAGA-3′
MITF:	F:	5′-CTCGAGCTCATGGACTTTCC- 3′
	R:	5′-CCAGTTCCGAGGTTGTTGTT- 3’
SDHC:	F:	5′-TTGAGTGCAGGGGTCTCTCT-3’
	R:	5′-AACCAGGACAACCACTCCAG-3’
HIF2A:	F:	5′-TTGATGTGGAAACGGATGAA-3’
	R:	5′-GGAACCTGCTCTTGCTGTTC-3’
VHL:	F:	5′-CCCAGGTCATCTTCTGCAAT-3’
	R:	5′-ACATTTGGGTGGTCTTCCAG-3’
MAX:	F:	5′-GAACGAAAACGTAGGGACCA-3’
	R:	5′-TGCTGGTGTGTGTGGTTTTT-3’
HRAS:	F:	5′-CCAGCTGATCCAGAACCATT-3’
	R:	5′-ATGGCAAACACACACAGGAA-3’
RET:	F:	5′-GCTGCATGAGAACAACTGGA-3’
	R:	5′-GGGTGACAGGAAGACCTTGA-3’
Bip/GRP78:	F:	5′-TAGCGTATGGTGCTGCTGTC-3’
	R:	5′-TTTGTCAGGGGTCTTTCACC-3’
CHOP:	F:	5′-TGGAAGCCTGGTATGAGGAC-3’
	R:	5′-TGTGACCTCTGCTGGTTCTG-3’
ATF4:	F:	5′-AAGGCGGGCTCCTCCGAATGG-3’
	R:	5′-CAATCTGTCCCGGAGAAGGCATCC-3’
ATF6:	F:	5′-ACCTGCTGTTACCAGCTACCACCCA-3’
	R:	5′-GCATCATCACTTCGTAGTCCTGCCC-3’

## Discussion and conclusions

We presented a case of pheochromocytoma with novel somatic mutation in RET gene occurring in WS with germline mutation of PAX3 gene. Furthermore, we illustrate the upregulation of UPR markers under extremely high expression levels of proto-oncogene RET in her pheochromocytoma by analyzing her normal adrenal gland as a control. Modulation of UPR may have a potential to be an alternative therapeutic strategy in pheochromocytoma with this RET gene mutation if surgical intervention is difficult.

Based on our patient’s clinical symptoms, shown as dystopia canthorum without muscle skeletal anomalies, she was clinically categorized as WS1 [2, 10]. This is the first case showing the association of pheochromocytoma with either germline or somatic RET gene mutation and WS. The point mutations in PAX3 gene are shown in more than 90% of patients with WS1 [11]. It is known that wild type PAX3 positively regulates RET mRNA expression as a transcription factor in vitro [8]. Ile59 in PAX3 is evolutionarily conserved [11]. The prediction of the three-dimensional structure of PAX3 revealed that Ile59Phe could affect regulation of the binding ability of paired domain to corresponding DNA [11]. In our case, it remains unknown if mutated RET per se increased its expression, or the mutated PAX3 under the tumor microenvironments enhanced positive transcriptional regulation of RET [8]. Although it is rare, accumulations of the cases are waited.

The mutations of Asp631_Leu633delinsGlu in RET gene are novel and located on the cysteine-rich domain [5]. The domain is located in the extracellular part of RET, and is thought to be related to ligand-independent dimerization and activation of RET. In our case, the mRNA expression levels of HRAS was increased in pheochromocytoma. This may support that RET was not only highly expressed, it was also hyperactivated since HRAS is in the downstream of RET kinase cascade.

The patients with pheochromocytoma with positive RET mutation are reported to have about only four times higher levels of RET mRNA expression than those who do not have it [12]. We could minimalize bias by using the samples from the same patient, unlike previous studies which were based on comparison between different patients. Thus, this might allow more accurate analysis of the high expression levels of mRNA of activated RET than previous reports.

UPR is known to play an important role in several endocrine diseases such as diabetes, obesity, Wolfram syndrome, and isolated growth hormone deficiency type II [13]. UPR has been shown to play an important role in pathologies of tumor progression [14]. Among the cell-intrinsic sources of ER stress drivers in cancer, protein synthesis rates are shown to be enhanced by loss or dysfunction of the tumor-suppressing genes or overexpression of oncogenic genes [14]. Thus, due to basal high production and storage of secretory proteins plus tumor-specific ER stress drivers, endocrine tumors are speculated to be under the activated UPR state. Indeed, Moore et al. recently first reported that UPR markers were increased about 1.5–5 times more in the pancreases of patients with pancreatic neuroendocrine tumors (pNET) than those without it [15]. On the other hand, we have developed KIRA, a mono-selective inhibiter of IRE1α, a key UPR sensor protein, and demonstrated that the modulation of the UPR by KIRA could ameliorate disease state in diabetes model mice and other ER stress-related diseases [9, 16, 17]. Furthermore, others recently demonstrated that administration of KIRA could prolong the survival of the model mice with pNET [15]. Thus, our finding may support the evidence that the UPR could be activated and, its modulation could potentially be an alternative therapy for endocrine tumors.

As for a limitation, although we analyzed the expression levels of RET and other mRNAs mainly in pheochromocytoma and adrenal medulla, we cannot exclude the possibility that adrenocortical tissues are contaminated. Although we carefully collected the samples with the expert pathologist, it is technically difficult to completely discriminate the very thin adrenocortical tissue from pheochromocytoma and adrenal medulla. Further studies using advanced technique such as live microdissection could be a way to solve this problem.

In conclusion, we presented a case of WS1 with pheochromocytoma. She had a novel somatic mutation of RET gene in her pheochromocytoma combined with the germline mutation of PAX3 gene. UPR markers were increased with marked increase of proto-oncogene RET mRNA expression in her pheochromocytoma. We believe that this rare case helps in the understanding of the pathological background of pheochromocytoma and suggests that targeting UPR may be a candidate novel therapeutic strategy towards endocrine tumors.

## Data Availability

The datasets used and analyzed during the current study available from the corresponding author on reasonable request.

## Abbreviations

WS: Waardenburg syndrome
MEN2A: Multiple endocrine neoplasia type 2A
FMTC: Familial medullary thyroid carcinoma
ER: Endoplasmic reticulum
UPR: Unfolded protein response
PAX3: Paired box gene 3
CT: Computed tomography
MRI: Magnetic resonance imaging
pNET: Pancreatic neuroendocrine tumors
